# ASO Author Reflections: Changes in Coagulation Biomarkers and the Risk for Venous Thromboembolism After Cytoreductive Surgery and Hyperthermic Intraperitoneal Chemotherapy

**DOI:** 10.1245/s10434-021-09960-6

**Published:** 2021-05-14

**Authors:** Paul Dranichnikov

**Affiliations:** Department of Surgical Science, Uppsala University Hospital, Uppsala, Sweden

## Past

Coagulopathy and changes in coagulation biomarkers after cytoreductive surgery (CRS) and hyperthermic intraperitoneal chemotherapy (HIPEC) is recognized but few details have been studied;^1^ however, high plasma D-dimer on postoperative day 3 was a risk factor for postoperative venous thromboembolism (VTE) following gynecological cancer surgery.^2^ The reported risk for postoperative VTEs in patients with peritoneal surface malignancy is estimated as 5.6% within 60 days after surgery.^3^ The aim of this study was to investigate postoperative changes in coagulation biomarkers and their predictive ability for VTE.

## Present

The study revealed that the 6% incidence rate of symptomatic VTE within 6 months of CRS and HIPEC was similar to comparable abdominal cancer surgeries. Furthermore, the study results revealed that residual tumor at the completion of surgery, as well as elevated D-dimer on day 2, were independent risk factors for postoperative VTE.

## Future

D-dimer on day 2 and incomplete cytoreduction of CC2–3 may be useful clinical markers to identify patients at higher risk for VTE after surgery; this could identify patients in need of prolonged prophylaxis. Further research on thromboprophylaxis and on changes in post-HIPEC coagulation biomarkers is needed, particularly research investigating whether clinically relevant cut-off values for D-dimer may exist.
